# Development of person‐centred quality indicators for aged care assessment services in Australia: A mixed methods study

**DOI:** 10.1111/hex.13958

**Published:** 2024-01-02

**Authors:** Sandra Smith, Catherine Travers, Natasha Roberts, Melinda Martin‐Khan

**Affiliations:** ^1^ Centre for Health Services Research The University of Queensland Brisbane Queensland Australia; ^2^ School of Health and Rehabilitation Sciences The University of Queensland Brisbane Queensland Australia; ^3^ Centre for Clinical Research The University of Queensland Brisbane Queensland Australia; ^4^ STARS Education and Research Alliance, Surgical Treatment and Rehabilitation Service (STARS) The University of Queensland and Metro North Health Brisbane Queensland Australia; ^5^ College of Medicine and Health University of Exeter Devon UK

**Keywords:** administration, assessment services, older adults, person‐centred, policy, quality indicators

## Abstract

**Introduction:**

This study developed a proposed set of person‐centred quality indicators (PC‐QIs) for services that assess older adults' care and support needs to determine their eligibility to receive government‐funded aged care services in Australia. Individual proposed PC‐QIs amenability for change within current organizational structures were explored. Barriers and opportunities to adapt service elements of the aged care assessment service to better align with the intent of the proposed PC‐QIs were identified.

**Methods:**

A mixed methods study was conducted over five phases. A scoping review identified domains of quality for aged care services as perceived by older adults. Service elements of an aged care assessment service were mapped alongside quality domains informing key attributes of each quality domain. Self‐determination theory was used to formulate each proposed PC‐QI to align with key attributes and quality domains. Consultation with a consumer group enabled revision of the proposed PC‐QIs. A focus group with clinicians evaluated the amenability of each proposed PC‐QI for change and identified barriers and opportunities to better align service elements with older adults' perceptions of quality. Results were informed by qualitative and quantitative data from a structured focus group. Focus group discussions were audio recorded and subsequently transcribed verbatim. Qualitative data were analyzed using a deductive thematic approach by two independent researchers.

**Results:**

Twenty‐four proposed PC‐QIs were developed. Refinement to descriptors of the proposed PC‐QIs were made by the consumer group (*n* = 18) and all were affirmed as being amenable to change by aged care assessors. Barriers in meeting the intent of the proposed PC‐QIs were identified across five domains including: health care staff knowledge (18.7%; *n* = 3); clear communication (31%; *n* = 5); person‐centred approach (18.7%; *n* = 3); respect for client (18.7%; *n* = 3); and collaborative partnership with client (12%; *n* = 2). Participants made 21 recommendations. Of the five service elements in delivering an aged care assessment service, barriers in meeting the intent of the proposed PC‐QIs were identified at the intake and booking of an assessment and during the assessment.

**Conclusions:**

Recommendations identified provide assessment services guidance on ways to adapt service elements to better align with older adults' perceptions of quality.

**Patient and Public Contribution:**

Patients and carers were involved as collaborators in this project at the protocol stage which included participating in discussions regarding the refining and modification of the protocol, refinement of the proposed PC‐QIs, data collection forms and supplementary information for participants.

## 1 INTRODUCTION

Globally, the population of older adults is increasing, both numerically and as a percentage of the total population. It is predicted that by 2050 the percentage of the global population of people aged 65 years or older will rise to 16%, an increase from 10% in 2022.^1^ This growth trend is relevant to all health care systems that aim to deliver long term‐care that is sustainable, promotes healthy ageing and either maintains or improves an older adult's quality of life.^2^

Aged care in Australia is highly regulated.^3^, ^4^, ^5^ Like other countries, such as Germany, Japan and Switzerland, the nationally government‐funded ‘Aged Care Assessment Programme’ identifies the needs of older Australians to determine their eligibility for and facilitate access to Commonwealth‐funded support services. Clinicians (nursing and allied health) conduct these assessments and work within an Aged Care Assessment Team (ACAT) in Australia. They are highly specialized in the comprehensive assessment of an older adult's needs, including physical, medical, psychological, cultural and social dimensions of care. Delivery of these assessment services requires compliance with legislation and policy using mandated reporting systems.^6^ Organizational and management structures of individual ACATs differ across health service districts, largely due to funding, governance, organizational structures and internal processes. The monitoring of quality is inspection‐based and managed by a single centralized authority, that is, the Australian Government Department of Health and Aged Care.

Significant reforms have occurred in the provision of aged care services in Australia over the last decade,^7^, ^8^, ^9^, ^10^, ^11^, ^12^, ^13^ incorporating models from international systems to improve the system's sustainability while ensuring appropriate care to older adults.^8^ The Royal Commission into Aged Care Quality and Safety made five significant quality improvement recommendations relevant to regulation and legislation, and are summarized in Box 1.^13^

BOX 1. **Relevant recommendations from the Royal Commission into Aged Care Quality and Safety (2018)**

*Recommendation 1* included establishing a new Aged Care Act to provide an aged care system that is based on an older person's universal right to high quality and timely support and one which enables older people to exercise choice and control over the planning and delivery of the care they receive.

*Recommendation 2* specified the new Aged Care Act should list the rights of older people seeking and accessing care including their right to equal access and choice between services available.

*Recommendation 22* included the expansion of quality indicators in residential aged care, the development of quality indicators in home care (home care services are services provided to community‐dwelling people) and the development of a quality‐of‐life assessment for consumers in both residential aged care and home care.

*Recommendation 23* specified that the Australian Government should use the quality indicators for ongoing continuous improvement by implementing reporting and benchmarking of individual service provider performance.

*Recommendation 28* replacing the current Aged Care Assessment Program, which includes Aged Care Assessment Teams and Regional Assessment Services, with a single comprehensive assessment process, and that this new assessment process should promote self‐determination and autonomy of the older person.

While the development, implementation, and reporting of quality indicators (QIs) in aged care services in Australia was a positive step by the Royal Commission to ensure that quality services are provided, there was no specific recommendation for the development, implementation or reporting of QIs for the Aged Care Assessment Programme.

Currently, the quality of aged care assessment services in Australia is measured by a set of key performance indicators directed by the Australian Government, Department of Health and Aged Care. Quality management tools, including a client satisfaction survey (10 questions) are used to measure the quality of the service provided.^14^

Elements of aged care assessment services in Australia are broadly categorized into five areas including the intake and booking of the assessment, conducting the assessment, follow‐up after the assessment has been completed, formulation of the support plan summary and a final letter summarizing the outcome of the assessment and approval for government‐funded services. Each service element has several components which are described in Table 1.

**Table 1:** Service elements of an aged care assessment service.

Service element	Description
Intake/booking of assessment	Web and phone‐based referrals are received via the My Aged Care Portal. Referrals are prioritized using a priority matrix (low, medium, or high) which provides a timeframe in which an assessment is expected to be completed. Assessments are allocated to an assessor, and an administrator arranges the booking of an assessment, which can occur over the telephone or via a letter in the mail.
Conducting the assessment	Aged care assessor uses a comprehensive assessment framework guided by the National Screening and Assessment Framework.^15^ Assessments can be conducted in a variety of settings including a client's home, hospital and residential aged care facility. This assessment guides the assessor's decision as to the eligibility status of the older adult for government‐funded aged care services.
Follow‐up	Aged care assessors communicate with relevant stakeholders as required. This can include medical and/or allied health and nursing professionals to confirm current care needs of the client. Aged care assessors discuss the outcome of the assessment with the older adult and/or their family.
Support plan summary	Aged care assessors develop support plan summaries (written document summarizing the older adult's current situation, their identified support needs and eligibility decision for aged care services). The support plan summary is mailed to the older adult.
Final summary letter of approval	Administrative staff use a Commonwealth government template outlining the older adult's approval of eligibility for specific aged care service types. This letter is mailed to the older adult.

Home care QIs (HC‐QIs) such as the second‐generation HC‐QIs developed by an international research network (interRAI) are currently used by organizations who deliver home care services. These indicators, derived from the Community Health Assessment and the Home Care Assessment,^16^ measure the functional (e.g., Cognition, Activity of Daily Living), Clinical (e.g., falls, pain), social (e.g., Caregiver distress) and utilization (e.g., hospital use) aspects of care.^16^ While these QIs can be used to measure the delivery of home care services, the domains measured do not align with the elements of an assessment service that determines an older adult's eligibility for access to home care services. For service elements such as assessment of eligibility, the perspectives of what older adults perceive to be quality service is very limited.^17^

The lack of quality domains that align with elements of assessment services that determine eligibility has been identified in an international scoping review.^17^ This international scoping review explored elements older adults considered to be important in the provision of community aged care services given the dearth of literature on assessment for eligibility. This research identified five client‐perceived quality domains for community aged care services, including (i) staff knowledge; (ii) respect for clients; (iii) a person‐centred approach; (iv) a collaborative partnership with a client; and (v) clear communication.^17^ Additionally, this scoping review identified the key attributes that defined each of the quality domains.

These findings highlight the need for person‐centred QIs (PC‐QIs) for assessment services that assess an older adult's eligibility for aged care programmes, to enable measurement of the provision of service elements to ensure that they not only meet legislation, policy and practice (inclusive of an organization's structure) but align with the perceptions of older adults who are receiving these services.

## 2 OBJECTIVE

The objectives of this study were to:

1. Develop a set of proposed PC‐QIs that measure the quality of the aged care assessment service from a client's perspective.
2. Identify the amenability for change of each proposed PC‐QIs in the current organizational structures of the aged care assessment service.
3. Explore barriers and opportunities for change to incorporating the proposed PC‐QIs into aged care assessment services from the perspective of aged care assessors.

This study is an analysis of qualitative and quantitative findings that aimed to answer the following research question:

1. Are the proposed PC‐QIs amenable to change and what do clinicians perceive are the barriers and opportunities for change to incorporating the proposed PC‐QIs into assessment services delivered by local health services in accordance with current organizational and structural processes?

### 2.1 Method

This study forms part of a multiphase research programme which aims to develop a set of PC‐QIs that measure the quality of the aged care assessment service from the client's perspective. This mixed methods study focussed on the development of a set of proposed PC‐QIs that will then inform the final stage of the multiphase research programme, which is outside the scope of this paper. This study involved a scoping review of the literature,^17^ consultation with a patient and carer advisory board and a focus group with aged care assessors from the largest public health service in Australia. Focus group participants were presented with the 24 proposed PC‐QIs describing older adults' perceptions of a quality community aged care assessment service and were asked to vote (yes/no) as to whether each proposed PC‐QI was amenable to change within the current organizational context. They were also asked to discuss barriers to incorporating the proposed PC‐QIs within the organizational context to achieve services that align with the perceptions of quality identified by older adults and formulate potential recommendations to address the identified barriers. The Consolidated Criteria for Reporting Qualitative Research guidelines were used to report our methodological approach (Appendix S1).

### 2.2 Ethical approval

This study was approved by the Royal Brisbane & Women's Hospital Human Research and Ethics Committee (project number HREC/2022/QRBW/82417) and ratified by The University of Queensland research ethics and integrity unit (project number 2022/HE001183). Governance approval was granted from the Metro North Hospital and Health Service Community & Oral Health Directorate, Queensland.

### 2.3 Setting

All focus group participants were recruited from the Metro North Aged Care Assessment Service, located in Brisbane, Queensland—part of Australia's largest and most diverse public health service. All participants were known to each other, and most were known to the research investigator (who previously worked as an aged care assessor at the service).

### 2.4 Recruitment of participants

Six ACAT assessors, identified by the service manager, consented to participate in one 90‐min face‐to‐face focus group which took place on 7 November 2022 at the participants' workplace. The focus group facilitator provided written information to the service manager outlining eligibility criteria for participants. The service manager approached eligible staff to invite their participation. To be eligible, participants must have successfully completed the ‘My Aged Care’ statement of attainment level four, aged care assessment training and/or aged care delegate training, which are mandatory requirements of the Australian Government, Department of Health and Aged Care for all aged care assessors.

### 2.5 Development of proposed PC‐QIs

The set of proposed PC‐QIs were developed across four phases and are outlined below.

*Phase 1*: Five *quality domains* and their corresponding key attributes identified by clients^17^ were used as the foundation for developing the proposed PC‐QIs (Table 2). This aimed to ensure the client's perspective of what a quality service looked like, was the key domain* in each proposed PC‐QI.

**Table 2:** Proposed person‐centred quality indicators used to inform focus group questions.

Domain*	Key attributes (title)	Proposed PC‐QIs (Phase 3)	Aged care assessment service element^	Self determination theory construct^#^	Advisory board review^€^
Health care staff knowledge	Heath care staff knowledge	1. During my assessment interview, I received information about the aged care assessment process which gave me confidence in the health care staff's knowledge.	Conducting the assessment	Relatedness; competence	Change to ‘health care staff’
	Health care staff clarity	2. During my assessment interview, I could understand what the assessor said to me.	Conducting the assessment	Competence	Simplification of wording
Respect for client	Respect during booking	3. At the time of booking my assessment, my cultural and/or religious preferences were respected.	Intake/booking of assessment	Relatedness	Simplification of wording, reorder sentence structure
	Respect during assessment interview	4. During my assessment interview, my cultural and/or religious preferences were respected.	Conducting the assessment	Relatedness	Reorder sentence structure
	Dignity during booking	5. At the time of booking my assessment, I was treated with dignity and respect.	Intake/booking of assessment	Relatedness; autonomy	Simplification of wording, reorder sentence structure
	Dignity during assessment interview	6. During my assessment interview, I was treated with dignity and respect.	Conducting the assessment	Relatedness; autonomy	Grammatical correction
	Support during assessment interview	7. During my assessment interview, I was supported to raise any concerns about getting the help I need.	Conducting the assessment	Autonomy; competence	
	Equitable booking process	8. At the time of booking my assessment, I was treated as an equal partner in the decision‐making process.	Intake/booking of assessment	Autonomy; competence	Grammatical correction, clarity of ‘equal partner’
	Equitable assessment interview	9. During my assessment interview, I was treated as an equal partner in the care planning process.	Conducting the assessment	Autonomy; competence	Grammatical correction, clarity of ‘equal partner’
	Respectful written information	10. Written information I received explaining my care needs reflected dignity and respect.	Final summary letter of approval	Relatedness	
	Accuracy of support plan	11. The support plan summary I received reflected my needs.	Support plan summary	Relatedness	
	Appropriate written information	12. Written information I received acknowledged the importance of culture and spiritual preferences.	Final summary letter of approval	Relatedness	
Person‐centred approach	Appointment convenience	13. My assessment appointment was scheduled at a time that was convenient to me.	Intake/booking of assessment	Autonomy; relatedness	
	Informed about support person	14. I was advised I could have a support person attend my assessment appointment if I so desired.	Intake/booking of assessment	Autonomy; competence	
Collaborative partnership with client	Involved in decisions at interview	15. During my assessment interview, I was provided with opportunities to make decisions about my care needs.	Conducting the assessment	Autonomy; competence	
	Adequate time for discussion	16. During my assessment interview, I had adequate time to talk with the assessor.	Conducting the assessment	Autonomy; relatedness	Simplification of sentence, grammatical correction
	Adequate time for decision making	17. During my assessment interview, I had adequate time to make decisions.	Conducting the assessment	Autonomy; competency	Reorder of sentence structure, grammatical correction
Clear communication	Responsibilities of aged care assessment team explained	18. During my assessment interview, the assessor explained the responsibilities of the aged care assessment team.	Conducting the assessment	Relatedness	
	Clear explanations by assessor	19. After my assessment interview was completed, the assessor explained what the next steps were.	Follow up	Autonomy; relatedness	Grammatical correction
	Knowledge of next steps	20. After my assessment interview was completed, the assessor explained what I was expected to do next.	Follow up	Autonomy; relatedness; competency	
	Contact information provided	21. If my care needs change after my assessment has been completed, I know who to access for assistance.	Follow up	Autonomy; relatedness; competency	
	Eligibility of care explained	22. I know what care I am eligible to access.	Conducting the assessment	Competency	
	Access to care explained	23. The assessor explained that an aged care assessment delegate would determine what aged care assessment type I had been approved to access.	Conducting the assessment	Relatedness	Recommendation to include this QI
	Easy to understand written information	24. Information I was given at my assessment interview was easy to understand.	Conducting the assessment	Competency	
Three subquestions asked in relation to each proposed PC‐QI					
(a) Is the proposed PC‐QI amenable to change? (Yes/No) (b) What are the barriers to incorporating the proposed PC‐QIs into local aged care assessment services?; and (c) What changes could be implemented within the current organizational context of the aged care assessment service, to address these barriers?					

Abbreviation: PC‐QI, person‐centred quality indicators.

*Phase 2*: The service elements of an aged care assessment service were mapped alongside the five quality domains identified in phase one. This informed the development of the key attributes (title) of each proposed PC‐QI (Table 2; ACAT service element^).

There are five distinct service elements that older adults experience when they interact with aged care assessment services (Table 1).

*Phase 3*: Self‐determination theory (SDT) was used as a framework to guide the development of the proposed PC‐QIs (Table 2; SDT^#^). This was done by ensuring the definitions of the three constructs of SDT, autonomy (sense of choice), competence (feeling good about what you can do) and relatedness (feeling connected to other people) were reflected in the proposed PC‐QIs. Replacement of the current aged care assessment programme with a single comprehensive assessment service that promotes self‐determination and autonomy of the older person was recommended (recommendation 28) by the Royal Commission into Aged Care Quality and Safety.^13^ To align with future directions of the aged care assessment programme, the use of SDT was seen as an integral aspect in the development of the proposed PC‐QIs. SDT defines three basic psychological needs of autonomy, competence and relatedness and argues that a person's satisfaction of these psychological needs promotes wellbeing and strengthens their inner resources which in turn strengthens their resilience.^18^ SDT is considered a general theory of human motivation and has been used in survey research, clinical trials and experimental studies in health.^19^ Kofi Osei‐Frimong^20^ investigated patient participatory behaviours in health care service delivery based on SDT which illustrated the importance of service providers understanding a participant's needs and motivations during a service encounter, to influence the nature of their participation, which in turn could improve the value and outcome of services delivered.

*Phase 4*: Patient and public involvement. The 24 proposed PC‐QIs were presented to the evaluating quality of care (eQC) Patient and Carer Advisory Board. This group provided feedback on readability and amendments were made accordingly (Table 2; Advisory board review^€^). The eQC Patient and Carer Advisory Board is a collaboration of patients and carers established to inform the embedding of partnerships between lived experience experts and researchers. Its aim is to ensure a consistent focus on participant collaboration across all stages of quality‐of‐care research including the planning, delivery, monitoring and evaluation phases of research. The eQC Patient and Carer Advisory Board is led by researchers based at the Centre for Health Services Research at The University of Queensland and is located at the Princess Alexandra Hospital in Brisbane, Australia. The Board has eight members of the public and includes persons living with dementia, carers or former carers of people living with dementia and, people with an interest in dementia/dementia services. The board was involved as collaborators in this project at the protocol stage which included participating in discussions regarding the refining and modification of the protocol and the proposed PC‐QIs, data collection forms, and supplementary information for participants.

*Development of structured focus group questions (questions asked of ACAT assessors to [a] determine if each proposed PC‐QI is amenable to change within the current organizational context [b] identify barriers and [c] generate recommendations to address barriers)*.

The semi‐structured questions for the focus group were developed using the proposed set of PC‐QIs and aligned with objectives two and three of the study.

Each proposed PC‐QI developed in Phases 1–4 had three subquestions:

1. Do you think the proposed PC‐QI is ‘amenable to change’? (Yes/No response) (*n* = 24).
2. What are the barriers to incorporating the proposed PC‐QI into local aged care assessment services? (*n* = 24).
3. What changes could be implemented within the current organizational context of the aged care assessment service, to address these barriers? (*n* = 24) (Table 2; proposed PC‐QIs).

A total of 73 focus questions including one introductory question, 24 questions requiring a yes/no response, and 48 open‐ended questions were developed (Table 2). Two members of the eQC Patient and Carer Advisory Board self‐nominated to participate in separate telephone interviews with the focus group facilitator, to trial the focus group questions. Feedback was invited regarding the facilitator's ability to conduct the group impartially and borne in mind when conducting the actual focus group.

### 2.6 Face‐to‐face focus group discussion

A face‐to‐face focus group was facilitated by the first author who is an occupational therapist who specializes in aged care. She worked as an aged care assessor in the Metro North ACAT from 2019 to 2020, has a sound understanding of the aged care assessment service elements and has completed training in qualitative methods. Five of the six participants were known to the facilitator through her previous work; however, participants were independently recruited by the service manager and the facilitator had no knowledge of their participation before the commencement of the focus group. The facilitator's previous roles most likely contributed to her having a greater awareness of the barriers and recommendations that may have been raised and discussed by participants. Accordingly, the facilitator briefly introduced the study and clearly explained that her role was to facilitate (and not influence) discussion before the formal process began. The group comprised six participants and the discussion took approximately 90 min.

At the focus group meeting each participant was provided with two paper‐based data collection sheets, one for demographic information and one to record their response (yes/no) to the amenability for change of each proposed PC‐QI. Information in the participant information sheet was presented verbally, including the recording and transcription of the focus group discussion, and anonymity of responses. Participants were advised that they could choose to cease participating in the focus group at any time. Before commencing the focus group discussion, the facilitator provided background information about the research project informing the development of the focus group questions. Instructions were verbally provided to the group, including an opportunity for questions before the process formally began.

Working definitions were agreed upon for the focus group discussion. The definition ‘Amenable to change’, was defined as: the outcome of the proposed PC‐QI measuring a client's ‘quality experience’ can be influenced and/or changed by modification of an ACAT service element that can be changed at the local level. The ACAT service element identified requiring change does not rely on systems and/or processes that are governed by controls that exist outside the influence of the local service itself.

Before proceeding to identifying influencing factors (i.e., barriers and potential strategies), assessors were asked to identify examples of legislation, policy and processes that could not be changed at the local service level, promoting a robust discussion between participants and further clarification of things that were outside the influence of the local ACAT Service. All participants acknowledged they were familiar with the aged care assessment quality framework which was designed to assist organizations to deliver a consistent approach of high‐quality My Aged Care assessments.^14^ Participants were presented with each proposed PC‐QI and their associated sub‐questions. Participants were first asked to vote (yes/no) as to whether the proposed PC‐QI was amenable to change and record their vote on a fit‐for‐purpose record form. They were asked not to discuss their votes with the group. As a group, participants were asked to discuss barriers in meeting the proposed PC‐QI (client's perception of service quality), and recommendations that could be implemented at a local level to address these barriers. The focus group was audiotaped and transcribed verbatim. Participant voting sheets were collated by the facilitator at the completion of the focus group.

### 2.7 Data collection

Participants completed a paper‐based data sheet with basic demographic data including their gender, age and number of years of practice as an aged care assessor and recorded his/her responses regarding each proposed PC‐QI's amenability for change on the record form.

The qualitative data transcribed from the audiotaped recording of the group discussion of barriers and potential strategies in meeting the objectives of each proposed PC‐QIs and the quantitative data collected from participants' voting sheets comprised the preliminary data for analysis.

## 3 RESULTS

### 3.1 Demographic data

Five of the six participants were female, and all were aged between 31 and 65 years. Collectively, participants had 78 years of experience working as an aged care assessor, with three assessors having 17–25‐years' experience.

### 3.2 Quantitative data

Six participants recorded 24 votes each (yes/no, *n* = 144) in response to the amenability of each proposed PC‐QI for change in the context of current structures. Twenty‐three of the 24 proposed PC‐QIs received a 100% yes vote (*n* = 138) affirming their amenability for change. One scored an 80% yes vote (*n* = 5) affirming its amenability, and a 20% No vote (*n* = 1). While aged care assessors identified barriers in meeting the intent of the proposed PC‐QIs, solutions to adapt service elements at the local level to meet the proposed PC‐QIs' intent were identified. Collectively, the small group of professionals (*n* = 6) affirmed all 24 proposed PC‐QI's amenability for change.

### 3.3 Qualitative interviews

Qualitative data were transcribed by the first author and initially displayed using participant responses word‐for‐word against each proposed PC‐QI. The deductive version of thematic analysis was then used to interpret the data and data were presented into the following coded categories: (1) aged care assessment service element (Phase 2); (2) quality domain (Phase 1); (3) barriers identified (mapped to quality domains representing each proposed PC‐QI); and (4) recommendations made by participants to address barriers. The intent was not to derive themes from the data, but to organize data into categories (as described above) in accordance with a deductive thematic approach. The data were then independently reviewed by a second researcher.

A total of 21 recommendations were made by participants to adapt aged care assessment service elements to better align with the proposed PC‐QIs' intent, that align with older adult's perceptions of quality, and that meet legislation, policy and organizational processes (Table 3). Recommendations were made for three out of the five aged care assessment service elements (intake and booking of assessment, assessment and follow‐up) across all five quality domains. Our reporting adheres to the standards for reporting qualitative research.^21^

**Table 3:** Barriers and recommendations in meeting quality domains aged care clients perceive as important across aged care assessment processes.

Aged care assessment service elements	Proposed quality domains	Barriers identified against quality domains	Recommendations to address barriers	Corresponding proposed PC‐QI
Intake/booking of assessment	Respect for clients.^a^ Person‐centred approach.^b^ Clear communication.^c^	Client's cultural and/or religious preferences not always considered.^a^ Communication between intake and administration staff not always consistent.^a^^,^ ^c^ Clients not always provided a choice of the time of assessment or the mode of delivery of assessment.^b^ Appointment times often mailed out to clients—limiting direct communication with clients.^b^ Clients not consistently advised they can have a support person present at the assessment if preferred.^b^	Cultural background and religious preferences, including the client's preference of using an interpreter service versus family member should be discussed. Clear understanding of and communication between intake and administration staff, of a client's cultural and religious preferences. Clear and accurate documentation of a client's cultural and religious preferences and preference for support person. Suitability of assessment time confirmed with client (option to offer appointments outside of scheduled times). Clients contacted by telephone to book assessment time. Information on waiting times provided Client's offered choice of mode of delivery of assessment, and choice documented once confirmed. Clients advised they can have a support person present at the assessment.	3‐4; 8; 13‐14; 18
Conducting the assessment	Respect for clients.^a^ Clear communication^c^ Collaborative partnership with client.^e^ Health care staff knowledge.^d^	Aged care assessment process is complex for clients to understand.^c^^,^ ^d^ Large volumes of assessment information provided to clients.^c^^,^ ^d^ Clients not provided with information about other clients' experience of aged care services.^e^ Clients not consistently provided opportunities to raise their concerns.^a^ Clients not always provided with adequate time to speak with the assessor.^e^ Clients and families don't always recall what they (clients') have consented to during the assessment.^c^ ^,^ d	Provide clients information about the assessment process before the assessment taking place. Provide information that is easy to read and limit the use of acronyms. Describe aged care services in accordance with Commonwealth government colour coded information booklets. For example, purple book = home care package. Develop easy read handout sheets that take into consideration clients with a cognitive impairment, those who have a decision maker and those from different cultural backgrounds. Easy read handout sheets could be used by assessors as a visual aid when explaining the aged care assessment process to the client/family. Provide information to client's that reflects their individual situation. Develop a one‐page simple flow chart providing information to clients on what to expect during the assessment, and what happens next. Assessors to show clients video resources available on the My Aged Care Website that describe a service experience from a client's perspective. Assessors should be appropriately skilled and trained at providing a more flexible approach to the assessment process, to accommodate different cultural backgrounds and clients who have communication and/or cognitive difficulties. Time allocated to assessors to complete the assessment should reflect the complexity of the client's situation. The use of allocating clients a ‘window of time’ for the assessment could be used to accommodate for assessor delays in assessments. Allocating assessments to accommodate the geographical area of clients could be used to reduce travel time and increase assessors' availability to clients. Clients should be provided with a copy of their ‘Application for care under the Aged Care Act 1997’.	1–2; 7; 9; 15–17; 22–24
Follow‐up	Clear communication^c^	Clients not consistently provided with clear information.^c^	Develop a one‐page flow chart advising clients of the next steps in the aged care assessment process.	19–21

Abbreviation: PC‐QI, person‐centred quality indicators

^a^ Respect for clients.

^b^ Person‐centred approach.

^c^ Clear communication.

^d^ Health care staff knowledge.

^e^ Collaborative partnership with client.

Direct quotes from focus group participants during discussion of aged care service elements are detailed in Table 4.

**Table 4:** Direct quotes from focus group participants.

Aged care assessment service element	Participant quotes
Intake and booking of assessment	(Participant) ‘The AOs (Administration Officer) make the bookings for the assessments, and they can certainly discuss aspects of the persons cultural background and religious preferences and whether they are comfortable with an interpreter for example, when they are making the booking. Some people are not comfortable with interpreters and prefer family to be there … you know … some people are not comfortable with interpreters depending on their community, and this often depends on what part they play in the community. This is often a cultural thing. Maybe the son is the decision maker for cultural reasons, and they don't want an interpreter for that reason’. (Participant) ‘For some people interpreters used may be known to them, and for some, they don't want to share this private information with other people from their culture’. (Participant) ‘I am wondering however if the AO does in fact ask the question when they are booking the assessment (spiritual and cultural preference of the client) … it could be that some AO's do and some don't. We wouldn't know if this was consistent’. (Participant) ‘Actually, the intake officer generally speaks to the client first, so I wander if this is something that the intake officer should be taking into consideration’. (Participant) ‘The intake officer generally emails the AO to give them the information about booking the assessment. But if the AO doesn't know to book an interpreter (to cater for a cultural preference), then they don't know what they don't know. There should be appropriate collection of information at intake really, and also adequate handover so we make sure we are meeting the clients' preferences’. (Participant) ‘One would hope that they would be asked if we can give you this time for your assessment, is it suitable for you? That they are informed about waiting times in terms of when the ACAT (aged Care Assessment Team) assessment would take place. You know … they could also be offered a preference about doing the assessment over the phone versus face to face. We seem to be a bit more flexible with telehealth after COVID’ (Participant) ‘Well I would hope that they would be asked if they would like a support person … and whether the appoint time is appropriate for them’. (Participant) ‘This is a tricky one, I think. I mean it can sometimes be hard because our schedules and appointments are so full it is often hard to find a convenient time for the client’. (Participant) ‘Where I think it falls down potentially is when we're using letters to inform clients of their appointment dates and times rather than calling them over the phone and having a discussion about it’. (Participant) ‘I suppose I can call and reschedule if the appointment time doesn't suit me as the assessor, but it is then about the balance of power. The balance is out, the assessor has all the power and not the client’.
The assessment	(Participant) ‘The whole aged care system is really complex and confusing for them (clients). It would be good for clients to be provided with information relating to the whole system before the actual assessment takes place. Sometimes it takes a significant amount of time at the beginning of the assessment to just explain the system in the most simplistic way. We are only one part of the whole system. They often (clients) don't know when the Aged Care Assessment Service stops and something else starts (in the whole aged care system). This makes it difficult’. (Participant) ‘We use all of these words like CHSP (pronounced Chisp), HCP, RAS assessor and ACAT assessor, and each information booklet we give them (clients) refers to different things which makes it so confusing for them (clients)’ (Participant) ‘They often don't know when the ACAT stops and something else starts. That sometimes makes it even more difficult’. (Participant) ‘Yeah … I think they confuse us (ACAT) with RAS (Regional Assessment Service) assessors. So that's what's worried me with some of the assessments of the clients I have done’. (Participant) ‘Yeah … I think that the word assessment is used too much. Clients don't know if it is a RAS assessment or an ACAT assessment, they just hear the word assessment. The confusion then continues, and when we go and do the ACAT assessment the client wonders what we are doing as they think they have already had an assessment (referring to them having had a RAS assessment previously) because they are already receiving services from a provider. They think that the services they are receiving are their package (services provided as a home care package for clients with medium to high care needs) and this is so confusing for them to understand’. Participant ‘Sometimes, with some clients, you need to go slowly. Really slowly to help them understand they can trust you. That they can trust the service. Some clients don't understand what you tell them in that one assessment, so you need to have a follow‐up hone call or visit. This is something that we used to do, but we can't do this anymore’ (Participant) ‘yeah … okay … the communication style of the assessment, you know, asking them (clients) have you had information, for example, on the assessment process itself?” And it could be easy to read handout sheets that the assessor can give to the client. Maybe we could have a number of resources and tools to use’.
Follow‐up	(Participant) ‘I think they (clients) think we are case managers, and that we then manage their care package or their aged care services. They really don't understand that we only do the assessment, and that is where we fit into the whole aged care system’. (Participant) ‘I haven't looked at one of the approval letters in a long time, but does it say something down the bottom about who you can contact for assistance? I don't know how much control we have because its, you know, it's a Commonwealth template that is used … right?’ (Participant) ‘You know, we could actually have a flow chart or something we could give them (clients) to make it easier’. (Participant) ‘We are told not to give out our phone number to clients. They are just given the call centre number, so if they want to talk to us directly, they can't’.

### 3.4 Intake and booking of assessment

Participants reported the interface between the responsibilities of different members of the ACAT across intake and booking of assessment process steps as barriers to achieving a quality service that meets clients' perspectives.

This was further explored, and participants reported information regarding whether a client's cultural and spiritual preferences had been considered; whether clients had been informed they could have a support person during their assessment; and what information clients had been provided regarding the aged care assessment process, were not always documented. This was identified by participants as impacting the next service element (the assessment).

Participants discussed difficulties with scheduling of assessments at convenient times for clients, highlighting assessors are often required to follow a structured timetable to accommodate the volume of assessments that need to be completed. Participants explored the benefits of contacting clients by telephone to arrange appointment times in preference to sending an appointment time in the mail. Participants reported this provided for a more direct line of communication and facilitated a two‐way dialogue to accommodate a client's preference.

### 3.5 The assessment

Participants discussed the complexity of the aged care system. Consensus was achieved by the group that information provided at the time of assessment was often excessive and did not always provide the client with a clear understanding of the aged care assessment process. Participants also identified the need to modify aged care assessment information to accommodate people from different cultural backgrounds and those with communication and/or cognitive difficulties. Participants identified the overuse of acronyms by assessors when explaining the aged care assessment service and the need to simplify their language during the assessment process.

### 3.6 Follow‐up

Participants reinforced during the discussion that an aged care assessor does not take on the role of ‘case manager’ for individual clients. The group discussed their limitations in providing extensive follow‐up after completing the assessment and providing the client with the relevant information about what services they had been approved to receive. The current follow‐up process was discussed and collectively they agreed the approval letter mailed to clients could be reviewed to improve its readability for clients.

## 4 DISCUSSION

To the best of our knowledge, this is the first study that has developed a set of proposed PC‐QIs for services that assess an older adults' eligibility for government‐funded aged care services, affirmed their amenability for change in accordance with legislation, policy and organizational structures, and explored the barriers in meeting their intent when delivering aged care assessment services. This mixed methods study presents clinician identified strategies for local service delivery levels. The ability to address 24 evidence‐based proposed PC‐QIs were quantitatively described. While barriers were identified in meeting the intent of the proposed PC‐QIs across three of the five aged care assessment service elements, participants discussed modifications that could be made at a local level to address these barriers and to facilitate a change in practice to improve the quality of the aged care assessment service, as perceived by clients.

Investigation of clinician perspectives on the implementation of quality domains has been used in other studies. The views of clinicians and quality improvement experts were explored in one study with the aim of understanding the measurement, acceptability and feasibility of implementing a person‐centred care framework at a health care system level, to inform the development of PC‐QIs.^22^ This study used structure, process and outcome components of a person‐centred care conceptual framework that was previously developed using a narrative review of the literature on PC‐QIs.^23^ The development of this person‐centred conceptual framework was guided by perspectives from patients (inclusive of family caregivers and representatives), and describes key person‐centred domains.^23^ By comparison, the findings of this study provided clinicians' views on the measurability of a set of proposed PC‐QIs, what barriers exist in meeting the intent of such measures and the resulting local policy and practice recommendations for change.

The aged care assessment process for rural older Australians has been the focus of a previous study that looked at improving the effectiveness of the assessment service in rural Australia. That study interviewed practitioners who delivered services in rural Australia to identify the challenges they faced in providing an integrated assessment service. A particular focus of the study was the exploration of how services could maximize their resources, improve the efficiency of assessment processes, and how to improve client outcomes. The study reported that practitioners were committed to improving systems for their clients and recognized there was a need to improve communication processes and data systems. The latter, however, related to sharing data and improving communication processes between service providers only.^24^

By comparison, the findings of our study were obtained through focus group questions based on a set of proposed PC‐QIs reflecting what older people have described as being important qualities of a service experience and presenting them to clinicians to facilitate discussion. This methodology provided for a robust exploration that included the perspectives of not only clinicians, but also of older service recipients.

Recommendations for improving communication between the client/family receiving the service and providers of the service were a key finding of our study. How care decisions of people living with dementia are made and experienced was explored in a systematic review. The findings of this study concluded decisions were made in three overarching ways, including, being excluded, prior preferences taken into account, and current preferences respected. This study, however, did not provide any recommendations on how to include the preferences of people with dementia in care decisions, but rather concluded that informal decision‐making was complex.^25^

The development of basic resources and easy read printed handouts was one recommendation to facilitate improved communication with clients with a cognitive impairment and in situations where a substitute decision maker is involved. The intention of this recommendation was to better facilitate a shared decision‐making approach between the assessment service, client, and the shared or substitute decision maker at the time of the assessment. The use of decision aids has been shown to be effective for older adults by increasing their knowledge and risk perception, decreasing conflict and enhancing participation in the decision‐making process.^26^ The use of TalkingMats (low technology communication framework that uses simple pictures on a textured mat) as a decision aid for older adults with mild to moderate dementia has also been shown to help people with dementia clarify their thoughts and express their views as well as helping them to come to a decision about how they felt they were managing with daily tasks.^27^

Another key finding of our study was the significance of incorporating strategies to ensure a client is treated with dignity and respect when receiving aged care assessment services. Recommendations included the importance of understanding a person's cultural background and religious preferences, including whether the client was comfortable using an interpreter service, or preferred family members to be present at the time of assessment. Freeman et al.^28^ outlined barriers that reduce cultural respect when providing primary health care services to Aboriginal and Torres Strait Islander Australians, including the inability to provide flexible and timely interpreter services, complex language used by staff, and racism and discrimination displayed by staff. The study also found the use of external programmes that were developed in research projects that existed outside the population group for which they were intended were a significant barrier to reducing the gap in cultural respect. The findings of our study also identified complex language used by staff as a barrier, however, this was viewed by participants as being applicable to all client groups regardless of their culture or religious background. A study using semi‐structured interviews to understand preferences of patients in receiving information identified five themes including autonomy, fostering relationships, access, communication and minimal information needs. Each theme was subdivided into subthemes. Reference to older adults in‐sub themes included; autonomy as supporting the older adult to be involved when receiving medications and providing written information they could understand, which in turn promoted autonomy; communication for the older adult described as providing information in small chunks and using everyday language; and minimal information needs described as older adults' preference to receiving only minimal information.^29^ To facilitate the greatest success when communicating with older adults Robinson et al.^30^ recommends using the following key elements; allow extra time, avoid distractions, sit face‐to‐face, maintain eye contact, listen, speak slowly, clearly and loudly, stick to one topic at a time and simplify and write down your instructions. The narrative for using short simple words and sentences suggested not to use medical jargon or technical terms that an older adult had difficulty in understanding, which directly relates with the findings of our study. The findings of our study are also relevant to the aged care sector internationally. Aged care systems are universal such that regulation, financing, workforce, quality, standards and organizations are key components of aged care systems globally.^5^, ^6^ The population of older adults is increasing not only by number, but also as a proportion of the total population.^1^ The United Nations proclaimed 2021–2030 to be the ‘Decade of Health Ageing’,^31^ with the aim to: ‘change how we think, feel and act towards age and ageing; ensure that communities foster the abilities of older people; deliver person‐centred integrated care and primary health services that are responsive to older people; and to provide access to long‐term care for older people who need it’.^32^

The provision of aged care services that not only comply with regulation, funding and quality systems of governments around the world, but that are perceived as quality by the person receiving them aligns with the aim of the ‘Decade of Healthy Ageing’.

While this study used quality domains derived from an international scoping review on what clients perceived as a quality service to develop the set of 24 proposed PC‐QIs that informed the focus group questions, findings of the present study (barriers and recommendations) were identified by participants in only one hospital and health service district in Brisbane, Queensland. This can be seen as a limitation of the study as processes may differ across services. However, services that assess an older adult's long term care needs from countries around the world including Australia, Canada, Denmark, England, Germany, Japan, Republic of Korea, The Netherlands, New Zealand, Poland, Russia, Singapore, Sweden, Switzerland and the United States are delivered in accordance with government legislation, policy and funding.^5^, ^6^ It is anticipated that the findings from this study can be used by aged care assessment services not only in Australia, but in other countries around the world as a benchmark to identify possible barriers and recommendations in delivering an aged care assessment service that clients perceive as a quality service that is in keeping with legislation and policy. Future work could further explore the needs of specific client populations of individual countries (e.g., communication nuances across cultural groups), and an organizations' day‐to‐day operational management and internal processes that influence the delivery of the aged care assessment service. Validation of the proposed PC‐QIs from the client's perspective will be explored in the final stage of this research programme.

## AUTHOR CONTRIBUTIONS

**Sandra Smith**: Conceptualization; methodology; formal analysis; project administration; investigation; writing—original draft; writing—review and editing; visualization. **Catherine Travers**: Conceptualization; methodology; supervision; writing—original draft; writing—review and editing. **Natasha Roberts**: Conceptualization; formal analysis; writing—original draft; writing—review and editing; supervision. **Melinda Martin‐Khan**: Conceptualization; methodology; supervision; writing—original draft.

## CONFLICT OF INTEREST STATEMENT

The authors declare no conflicts of interest.

## Supporting information

Supporting information.

Supporting information.

Supporting information.

Supporting information.

## Data Availability

Data will be made available upon request and at the discretion of the authors.
