# Supplementary material for: Development of person‐centred quality indicators for aged care assessment services in Australia: A mixed methods study
Source: Health Expect. 2024 Jan 2;27(1):e13958. doi: 10.1111/hex.13958 (PMC10768856; doi:10.1111/hex.13958)
Supplement: Supplementary file 2 — Supporting information. [file HEX-27-e13958-s004.docx]

**THE AGED CARE ASSESSMENT PROCESS**

1.
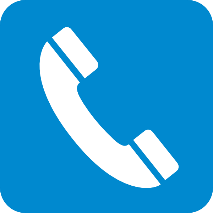
**REFERRAL REQUEST**

You, your family member, your doctor, a friend, or a health professional can ask for an aged care assessment.

**2.0** **ARRANGING YOUR ASSESSMENT TIME**


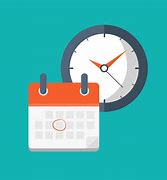
The administration staff from the Aged Care Assessment Team contact you, and/or your family member and arrange a day and time for the assessment to take place.

**3.0** **ON THE DAY OF THE** **AGED CARE ASSESSMENT**

An aged care assessor comes to your house and asks you some questions about what help you need. If there are restrictions in place because of COVID 19, the assessor might call you instead. Your family member can be with you to help you answer the questions.
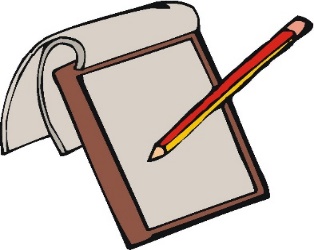


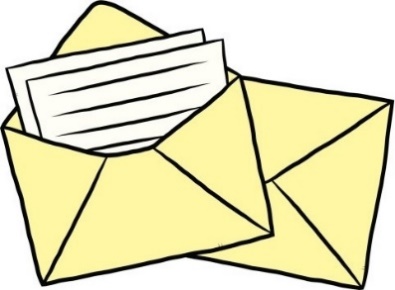


**4.0 RECEIVING THE SUPPORT PLAN SUMMARY**

Information is sent in the mail to you and/or a member of your family. This information tells you about what help you need with your everyday activities.


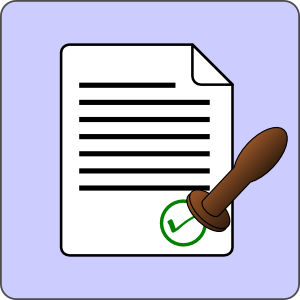
**5.0 APPROVAL LETTER**

A letter is sent in the mail to you and/or a member of your family. This letter gives you information on what government funded aged care services you are approved to access.
