# Supplementary material for: Development of person‐centred quality indicators for aged care assessment services in Australia: A mixed methods study
Source: Health Expect. 2024 Jan 2;27(1):e13958. doi: 10.1111/hex.13958 (PMC10768856; doi:10.1111/hex.13958)
Supplement: Supplementary file 3 — Supporting information. [file HEX-27-e13958-s001.docx]

**Easy read research information sheet**

This sheet is about the research you have been asked to participate in.


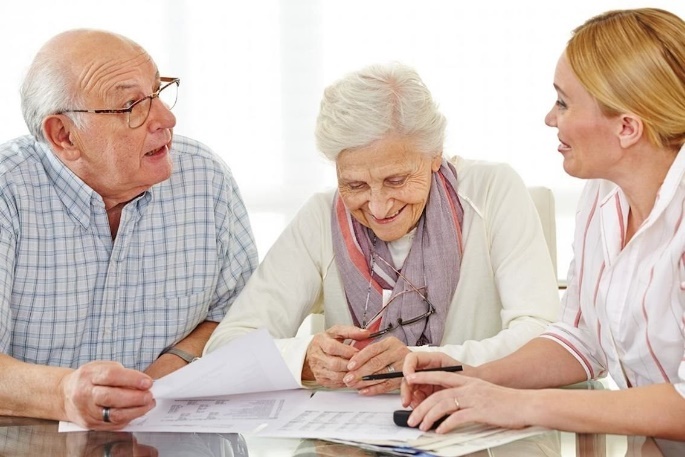


This research is about the aged care assessment process. You may or may not have recently experienced. It is about what you think was important about that experience.


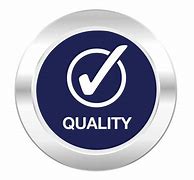


Quality is about receiving a good service.

It is important for the Aged Care Assessment Team to understand what you think a quality service is.


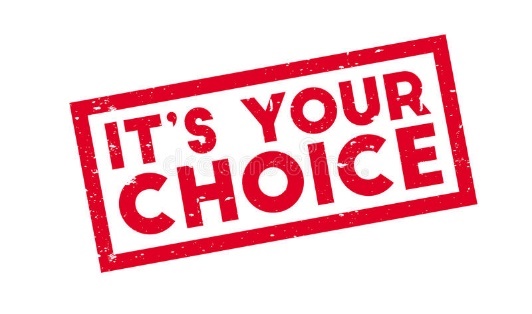


It is your choice if you would like to participate in this research.

*
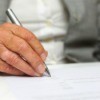
*

If you are happy to participate in this research, you will be asked to sign a form.


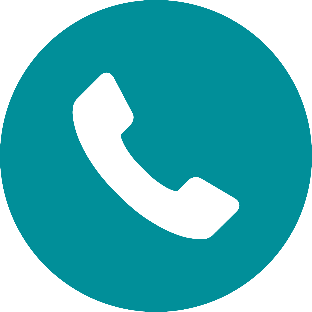


A researcher will call you to arrange a time to visit you at your house.


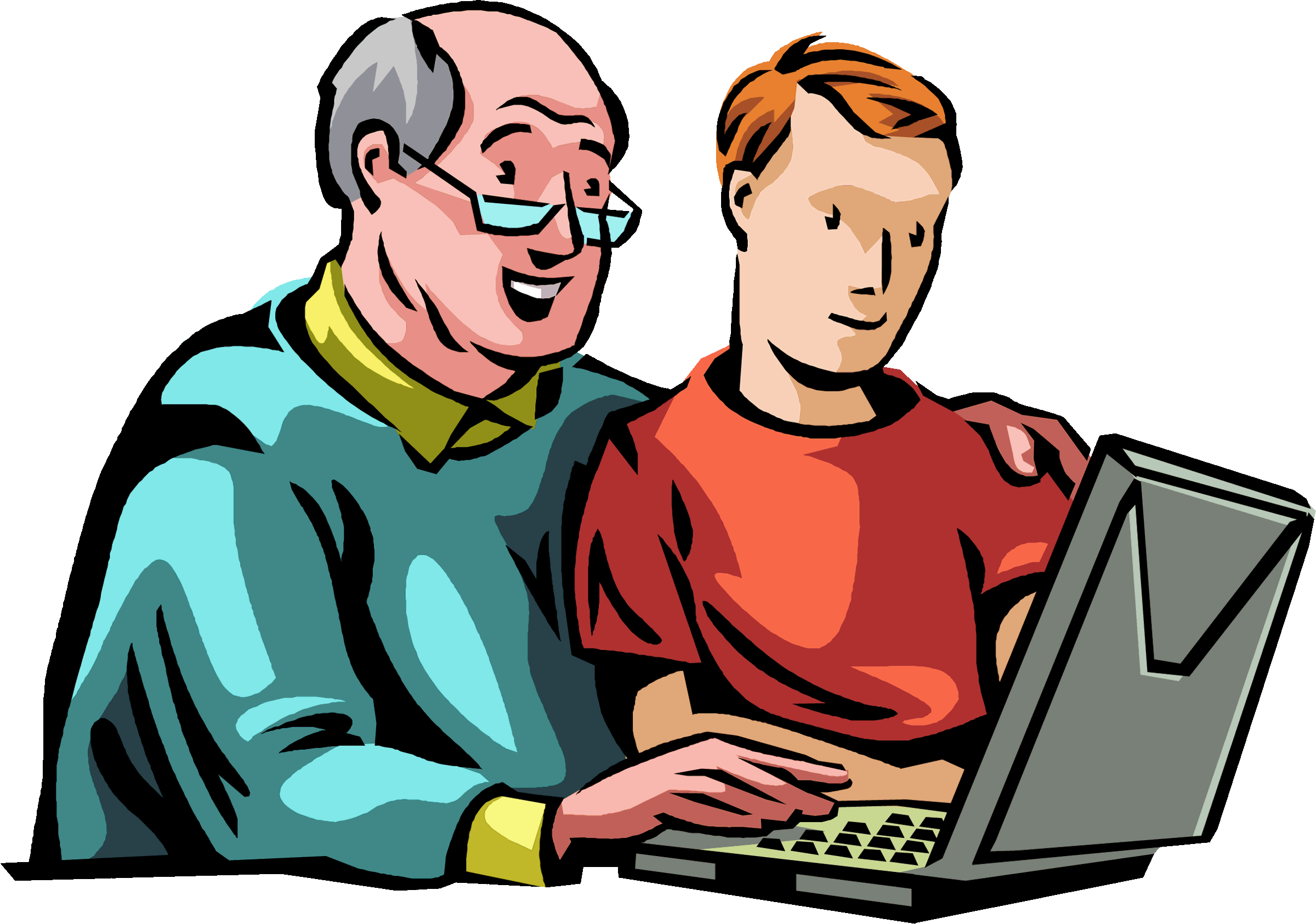


A member of your family, or somebody who supports you to make decisions can be with you to help you answer the research questions.


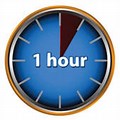
The visit at your house will take approximately one hour.

You can take as many breaks as you like if you get tired.


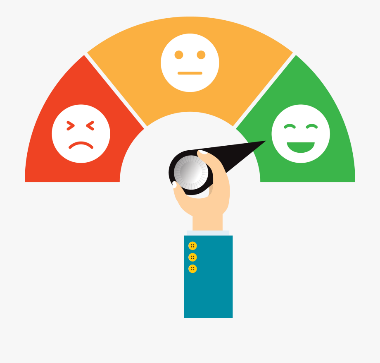


You will be asked some questions about what you think makes an aged care assessment a good experience for you.


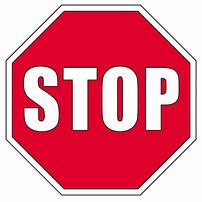
If you want to stop participating in the research at any time, you can.
