# Supplementary material for: Development of person‐centred quality indicators for aged care assessment services in Australia: A mixed methods study
Source: Health Expect. 2024 Jan 2;27(1):e13958. doi: 10.1111/hex.13958 (PMC10768856; doi:10.1111/hex.13958)
Supplement: Supplementary file 4 — Supporting information. [file HEX-27-e13958-s002.docx]

**How can the Aged Care Assessment Team help you?**

THE AGED CARE ASSESSMENT TEAM **GIVE YOU INFORMATION** ABOUT WHAT CARE IS AVAILABLE

The Assessment can take place:

1. **at your home**,
2. **in a hospital, OR**
3. **in a residential aged care facility**

**Access** to receive services under the Commonwealth Home Support Program

**Access** to receive the Short Term Restorative Care Program (time limited rehabilitation program provided in your home environment)

THE AGED CARE ASSESSMENT TEAM CAN **RE-ASSESS** YOUR CARE NEEDS IF YOUR CIRCUMSTANCES CHANGE.

THE AGED CARE ASSESSMENT TEAM **DETERMINE** IF YOU ARE **ELIGIBILE** TO **ACCESS AGED CARE**

**Access** to move into a Residential Aged Care Facility *permanently*

**Access** to move into a Residential Aged Care Facility *temporarily* (respite)

**Access** to receive a Home Care Package

THE AGE CARE ASSESSMENT TEAM **APPROVE** YOUR ACCESS TO AGED CARE
